# Nighttime sleep duration and hedonic eating in childhood

**DOI:** 10.1038/ijo.2015.132

**Published:** 2015-08-11

**Authors:** L McDonald, J Wardle, C H Llewellyn, A Fisher

**Affiliations:** 1 Department of Epidemiology and Public Health, Health Behaviour Research Centre, University College London, London, UK

## Abstract

**Background:**

Higher food intake is implicated in the elevated risk of obesity associated with shorter sleep in children, but the mechanisms driving higher intake are uncertain. Research in adults suggests that acute sleep deprivation affects brain reward systems, which increases responsiveness to palatable foods. However, there have been few studies addressing habitual sleep duration, and few in children, among whom the strongest associations with body mass index (BMI) are seen.

**Objective:**

The objective of this study is to test the hypothesis that shorter-sleeping children are more food responsive and explore the mediation of the relationship between sleep and weight by food responsiveness (FR).

**Methods:**

Participants were families from Gemini, a UK twin birth cohort, who had provided complete information on their children's sleep and appetite at age 5 years (*n*=1008). One child from each twin pair was randomly selected for analyses. Nighttime sleep duration was calculated from parent-reported bedtime and wake time, and categorised as shorter, adequate or longer according to age-specific reference values. FR was assessed with the Child Eating Behaviour Questionnaire. BMI s.d. scores (BMI-SDS) were calculated from parent-measured heights and weights using the UK 1990 reference data and were available for 494 children.

**Results:**

There was a significant linear association between shorter sleep and higher FR at age 5 years (*P* for linear trend=0.032), which was maintained after adjusting for age, sex, birth weight, maternal education and BMI-SDS. In the subset with BMI data at age 5 years, shorter sleep was associated with higher BMI-SDS (*P*=0.026) as expected. Testing for mediation by adding FR to the model attenuated the linear relationship to borderline significance (*P*=0.049), suggesting partial mediation.

**Conclusions:**

Shorter sleep in childhood is associated with higher FR, which may partly explain the association between shorter sleep and adiposity in childhood.

## Introduction

Short sleep in childhood has been shown to significantly raise the risk of overweight and obesity.^1, 2^ Evidence to date points to food intake rather than activity as the primary pathway. Studies in healthy adults in which nighttime sleep is restricted show increased energy intake and weight gain.^3, 4^ Epidemiological studies in children have identified an inverse relationship between sleep duration and energy intake.^5, 6^

Interest has now turned to the mechanisms through which shorter sleep affects food intake. Neuroimaging data show that sleep deprivation increases activity in brain reward centres in response to images of palatable food, as well as ‘desire to eat'.^7^ Experimental studies in adults, in the context of *ad libitum* energy intake, have also shown that controlled sleep curtailment increases preference and consumption of energy-dense foods without any evidence of change in metabolic signals.^3^ These observations suggest that short sleep affects food intake via hedonic rather than homeostatic processes.^8^

Most studies to date have investigated acute effects of sleep deprivation and there have been few studies examining effects on food responsiveness (FR) in relation to habitual shorter sleep. There are also no pediatric studies, despite this being the stage at which associations between sleep and weight are most consistent.^2^ One study in children aged 5–12 years showed that shorter sleep was associated with lower scores on a scale of ‘external eating', a construct with considerable overlap with FR, but associations between sleep and weight were not reported.^9^

In the present study, the primary hypothesis was that habitual shorter sleep at age 5 years would be associated with higher FR. In the subset of participants with weight data at 5 years, we tested the hypothesis that FR would partly mediate the association between sleep duration and weight. As a secondary analysis, we also examined whether sleep duration was associated with homeostatic eating (indexed with satiety responsiveness (SR)) to test whether the sleep–appetite associations was general rather than specific to FR.

## Subjects and methods

### Participants

Participants were from Gemini, a UK twin birth cohort. The Gemini study has been described in detail previously.^10^ Briefly, all families with twins born in England and Wales between March and December 2007 were contacted through the Office for National Statistics. The baseline sample of 2402 families represented 36% of all live twin births during this period. The present study used data collected in 2012, when the children were on average 5 years old. Data were from 1008 families who had provided complete information on their children's sleep and appetite at this age. To avoid clustering effects, one child from each twin pair was randomly selected for the analysis. Mothers who provided complete data were slightly older, more highly educated and more likely to be from a White ethnic background (all *P*'s <0.001).

At this age, complete height and weight data were available for only 494 (21% of baseline sample). Mothers who provided this information were more likely to be university educated, but there were no differences between the study sample and the sample with body mass index (BMI) data on any of the key variables for this analysis (sleep duration, FR and birth weight). Parents of participating families provided informed written consent. The University College London Committee of Non-National Health Service Human Research granted ethical approval.

### Measures

#### Sleep

Nighttime sleep duration was calculated from parent-reported bedtime and wake time at age 5 years. Sleep was then categorised as shorter (<11 h), adequate (11–12 h) or longer (>12 h) using age-specific reference values.^11^ This method is common in large population-based studies and has been validated using actigraphy in young children.^12^ In a subsample of 40 Gemini families, 1-week test–retest reliability of the sleep items was high (intraclass correlation: 0.89; 95% confidence interval: 0.76–0.95 for nighttime sleep duration). Categorising sleep has the benefit of allowing comparisons to be made with the sleep–weight literature and the previous sleep research in Gemini.^5, 13^

#### Appetite

We assessed FR with the FR scale of the Child Eating Behaviour Questionnaire. We also included the SR scale of the Child Eating Behaviour Questionnaire as an indicator of ‘homeostatic eating'. The Child Eating Behaviour Questionnaire is a validated 35-item instrument designed to assess a range of appetitive traits implicated in the regulation of body weight.^14^ The FR scale has five items assessing the degree to which a child expresses a desire for food, in particular in response to palatable food cues (for example, my child is always asking for food). The SR scale has five items assessing the degree to which a child tends to stop eating (or does not initiate eating) according to their perceived fullness (for example, my child cannot eat a meal if she/he has had a snack just before). Both FR and SR scales have good internal consistency and reliability, and have been validated against objective measures of food intake in childhood.^14, 15^

#### Body mass index

Gemini families were provided with electronic weighing scales and height charts when the children were 24 months and were asked to provide 3-monthly height and weight measures. Weight and height data included in the analyses were those collected closest to questionnaire completion. If the exact 5-year data were not available, data points within 3 months of this age were used. Weight and height data were available for 494 children at age 5 years. Age- and sex-adjusted BMI s.d. scores (BMI-SDS) were calculated using the UK 1990 reference data.^16^

#### Socio-demographic characteristics

Maternal education was assessed in the baseline questionnaire. Mothers reported on their highest level of education, which was categorised into lower (compulsory basic schooling), middle (some additional schooling or vocational qualifications) and higher (university educated). Birth weight was reported by asking parents to photocopy or transcribe health records.

### Analysis

Sleep and appetite data were normally distributed. Univariate analysis of variance models using tests of a linear association were used to compare FR and SR between the sleep groups. Analysis of covariance models were then used to test whether any observed effects were independent of age, maternal education, sex and birth weight. Models were also adjusted for BMI-SDS scores; however, doing so substantially reduced the sample size and adjusted models are presented with and without the inclusion of BMI-SDS.

Comparison of BMI-SDS between sleep groups was carried out using analysis of variance with polynomial contrasts testing for a linear association. Analysis of variance models were first run to examine the linear relationship between sleep duration and BMI-SDS. To test whether the sleep–weight relationship was attenuated by differences in eating behaviour, any trait significantly associated with sleep in previous models was entered into the analysis of covariance model predicting BMI-SDS. Hayes' bootstrapping PROCESS macro for SPSS was used to evaluate mediation.^17^

## Results

Participant characteristics are shown in Table 1. In total, 1008 children had complete sleep and appetite data at age 5 years (mean 5.2 years, s.d. 0.1). Average nighttime sleep duration was 11.48 h (s.d. 0.66 h), the average FR score was 2.84 (0.61) and the average SR score was 2.38 (0.76). BMI-SDS information was only available on 494 children, with a mean value of −0.20 (0.96).

Univariate and multivariate associations between sleep and FR are shown in Table 2. There was a significant linear relationship between nighttime sleep duration and FR, such that shorter sleep was associated with higher FR at age 5 years (*P* for linear trend=0.032). These associations were retained after adjusting for age, sex, maternal education, birth weight and BMI-SDS, the latter adjustment reducing sample size. There was no significant association between nighttime sleep duration and SR (*P* for linear trend=0.749).

The results of analysis of variance and analysis of covariance models predicting BMI-SDS in the subset of the sample with available weight data are given in Table 3. As expected, shorter nighttime sleep was associated with higher BMI-SDS (*P* for linear trend=0.026). The linear relationship was strengthened after adjusting for age, sex, birth weight and maternal education. To test mediation, we added FR into the analysis of covariance model predicting BMI-SDS. This model attenuated the linear association between sleep and BMI-SDS to borderline significance (*P* for linear trend=0.049). The Hayes' PROCESS add-in for SPSS demonstrated the mediation effect via FR was significant (−0.04 (0.02); 95% confidence interval: −0.09 to −0.01).

## Discussion

This study provides strong evidence for an association between habitually shorter nighttime sleep and higher FR in childhood. We also observed that higher FR could partly account for the linear relationship between sleep and BMI-SDS at age 5 years. In contrast, sleep duration showed no association with SR, a measure of homeostatic eating.

These findings support experimental work in adults, which has suggested that acute sleep deprivation influences ‘hedonic' rather than ‘homeostatic' pathways to food consumption.^3, 8^ The results are also similar to the one previous pediatric study, which found that shorter sleep was associated with higher external eating but not with emotional or restrained eating.^9^ External eating has conceptual overlap with FR in that both traits reflect the propensity to overconsume in response to palatable food cues.

Among adults, one previous study has shown that a tendency to disinhibited eating moderates the association between sleep and BMI, with a stronger relationship among adults who had higher disinhibited eating.^18^ Disinhibited eating reflects the propensity to eat opportunistically within an obesogenic environment,^19^ thus again having similarities to the FR scale of the Child Eating Behaviour Questionnaire. Furthermore, functional imaging studies have shown that sleep loss increases activation in reward centres of the brain, and subsequently food desire, in response to images of highly palatable foods but not in response to healthy food items.^7^ Together, these findings indicate that suboptimal nighttime sleep may specifically increase the salience of palatable foods, and consequently the drive to consume, within a permissive environment.

Although this study provides evidence that sleep may increase hedonic eating in children, more work is needed to show that this in turn drives overconsumption among shorter sleepers. Studies on dietary intake have shown that shorter sleeping children have more frequent eating occasions and more unfavourable dietary patterns, in particular a higher intake of energy-dense foods.^6, 20, 21^ These patterns of consumption characterise a kind of ‘hedonic overeating',^22^ where eating is responsive to food cues rather than due to impaired satiety processes.^8^ There is a need for longitudinal research to establish whether the relationship strengthens as children gain increasing autonomy over their eating behaviour and food environment, and whether factors such as food availability, accessibility and rules in the home environment influence susceptibility to weight gain. Given that parents largely control the food environment at this stage, this could have implications for interventions to prevent excess weight gain, for example, controlling exposure and access to palatable food items among children who experience difficulty sleeping, in particular at night when parents may be more inclined to feed-to-soothe.^13^

### Limitations

This study has some limitations that should be considered. The cross-sectional design means longer-term follow-up is needed to understand the temporal distribution of the relationship between sleep, FR and weight gain. Our sample included only children with complete data on sleep and appetite, and for the mediation analyses only a smaller sub-sample with BMI data, thereby excluding a considerable proportion of the Gemini sample. Children with complete data had mothers who were more likely to be university educated and from a White ethnic background, which limits the generalisability of the findings; hence, replication in a more diverse sample is required. Gemini children are leaner with respect to the UK 1990 reference values, reflecting the fact that twins tend to be born smaller than singletons.^23^ Although this could limit the generalisability of the findings, there is no strong reason to expect that the association between sleep and weight, and the factors that mediate this relationship, should differ between twins and singletons.

Parent-reported nighttime sleep is a limitation, although this method is common in larger population-based studies where objective measurements are not feasible. Encouragingly, the mean nighttime sleep duration in this sample is comparable to published reference values for children at age 5 years.^11^ Furthermore, calculating nighttime sleep from parent-reported bedtime and wake time has been validated against actigraphy in young children^12^ and might provide a better global representation of sleep behaviour than a few nights of objective recording.

## Conclusion

We show that shorter sleep at age 5 years is associated with higher FR but not with SR. Mediation analysis is consistent with the idea that FR is part of the pathway mediating the effect of shorter sleep on adiposity.

## Figures and Tables

**Table 1:** Participant characteristics given as mean (s.d.) unless otherwise stated

*N*	1008
Birth weight	2.46 (0.54)
Maternal education (%): low/mid/high	27.4/22.1/50.5
Sex (%): male/female	49.4/50.6
Nighttime sleep (h)	11.48 (0.66)

*Appetite*	
Satiety responsiveness	2.38 (0.76)
Food responsiveness	2.84 (0.61)
BMI-SDS^a^	−0.21 (0.96)

Abbreviation: BMI-SDS, body mass index s.d. score.

a Data available for *n*=494.

**Table 2:** ANOVA and ANCOVA model for associations between appetitive traits and nighttime sleep duration

*Nighttime sleep duration*	*<11 h*	*11–12 h*	*>12 h*	P *(linear trend)*
*Univariate models*				
Food responsiveness	2.53 (0.08)	2.36 (0.03)	2.35 (0.04)	0.032*
Satiety responsiveness	2.80 (0.07)	2.89 (0.02)	2.80 (0.03)	0.749

*Multivariate models*^a^				
Food responsiveness	2.55 (0.07)	2.36 (0.03)	2.35 (0.05)	0.022*
Satiety responsiveness	2.82 (0.06)	2.88 (0.03)	2.76 (0.04)	0.372

*Multivariate models*^b^				
Food responsiveness	2.51 (0.09)	2.32 (0.04)	2.28 (0.60)	0.038*
Satiety responsiveness	2.85 (0.08)	2.89 (0.03)	2.75 (0.05)	0.229

Abbreviations: ANCOVA, analysis of covariance; ANOVA, analysis of variance; BMI-SDS, body mass index s.d. score.

Data given as mean (s.e.). *P=0.05.

a Adjusted for age, sex, birth weight and maternal education.

b Adjusted for all covariates and BMI-SDS.

**Table 3:** ANOVA and ANCOVA models for association between sleep at BMI-SDS, adjusting for appetitive traits FR and SR

*Nighttime sleep duration*	*<11 h*	*11–12 h*	*>12 h*	P *(linear trend)*
*BMI-SDS*				
Univariate	0.01 (0.15)	−0.15 (0.05)	−0.35 (0.10)	0.026*

*Multivariate models*				
Model 1	0.05 (0.14)	−0.16 (0.06)	−0.36 (0.09)	0.015*
Model 2	−0.01 (0.13)	−0.16 (0.06)	−0.33 (0.08)	0.049*

Abbreviations: ANCOVA, analysis of covariance; ANOVA, analysis of variance; BMI-SDS, body mass index s.d. score; FR, food responsiveness; SR, satiety responsiveness.

Data given as mean (s.e.). Model 1 adjusted for age, sex, birth weight and maternal education. Model 2 adjusted for all covariates and FR. *P=0.05.
